# A Cross-Sectional Study: Are Myths on Cleft Lip and Palate Still Prevalent?

**DOI:** 10.7759/cureus.19579

**Published:** 2021-11-14

**Authors:** Sravya Turlapati, Sai Krishna, Korutla U Deepak, Baggialaxmi Kanagaraja, Kanaparthi A Gayathri, Divya Jahagirdar

**Affiliations:** 1 Public Health Dentistry, Government Dental College & Hospital, Hyderabad, IND; 2 Preventive & Community Dentistry, S.B. Patil Dental College, Bidar, IND; 3 Department of Public Health, University of Huddersfield, West Yorkshire, England, GBR; 4 Dental Surgery, Government Dental College & Hospital, Hyderabad, IND

**Keywords:** cleft lip & palate, high-risk pregnancy, birth defect, congenital birth defect, myths & taboos

## Abstract

Background

The etiology of cleft lip and palate (CL/P) remains largely unidentified. Evidence-based research shows a strong association with genetics, environmental factors, nutritional deficiency, smoking, alcohol, and drug misuse. Despite the increase in knowledge and widespread access to medical care beliefs contrary to science, folklores on CLP still occur in most developing countries.

Methodology

The study design was cross-sectional in nature and involved a sample of 136 parents of children with cleft lip and palate reporting to Smile Train Cleft Centers. It was conducted by using a self-structured questionnaire from December to March 2019.

Results

The highest recorded response was holding sharp objects, such as knives, scissors, or needles, during pregnancy (40.4%) and the least recorded response was for pregnant women going out on an auspicious day (3.7%).

Conclusion

The current study demonstrated that a majority of the parent’s socioeconomic status was upper lower class. Some parents still believe in the myths around the etiology of CLP despite the advances in medicine and technology.

## Introduction

Cleft lip (CL) and cleft palate (CP) are congenital deformities caused by unusual embryonic facial development in the course of intrauterine life. CLP is a major public health problem affecting 1.47 in thousand live births globally, and in India, the estimated incidence is around 0.25 to 2.29 per 1000 births [1]. In India, the calculated prevalence rate/100,000 was 33.27 for males and 31.01 for females [1-2].

The severity of the cleft and “the immediate impact followed by the long-range effects may influence the parent’s perceptions, reactions, and needs” [3]. CLP has a profound impact on the family system, as not only do parents have to adjust to the normal demands of parenthood, but they also have to deal with increased stresses and challenges resulting from the disability [4]. Most parents are often not prepared for a child with a facial cleft deformity and generally do not have the necessary knowledge to deal with the unexpected deformity [5]. They feel guilty and embarrassed, which is often intensified if either parent feels they did something wrong during the pregnancy to damage the baby.

Cultural factors are inherently involved in shaping maternal reactions to childbirth. Maternal birth reactions differ across cultures, particularly concerning children with cleft lip and palate. In developing countries where prenatal care is less advanced, a CL/P is usually unpredicted and families have faith in religion and folklore than medical explanations to explain the deformity. For instance, individuals in India who practice Hinduism believe that a CL/P is the result of sins from a past life [2]. Other religious and cultural beliefs regarding the causation of clefts include witchcraft, God’s will, and engaging in behavior associated with causal power. Responses to physical deformities are influenced significantly by beliefs and attitudes. Reactions to having a child with a cleft lip/palate are different across cultures and are likely influenced by the cultural beliefs surrounding the cause of CL/P. Cultural assumptions and beliefs about the cause of a CL/P can have a profound impact on the affected individual and his/her family [3]. As the studies in this regard are very limited, the identification of these issues is neglected and thus a great concern. Hence, this study was conducted to investigate the myths around the etiology among the parents of cleft lip and palate.

## Materials and methods

A cross-sectional study was conducted among parents of children with CLP at a Smile Train Cleft Center in Hyderabad city, Telangana, India, to assess their extent of social stigma and myths surrounding the etiology of CLP. The study consisted of parents of children aged up to 15 years with cleft lip and cleft palate at Smile Train Centers in Hyderabad city, Telangana.

A standardized Affiliate Stigma scale developed and validated by Winnie Mak in 2008 was selected for the study. The original English version of the questionnaire was translated to the local language by an oral health professional well-versed in both languages and validated.

The myths scale was constructed by the investigator based on the previous literature. Seven questions related to myths and beliefs were prepared. The content validity and face validity were established to evaluate if the questions effectively captured the topic under investigation.

A pilot study was conducted on 33 parents of these children to check the feasibility and to note any difficulties encountered during the data collection. The questionnaire also included information on socio-demographic variables using the Kuppuswamy socioeconomic status scale, family history, and the order of the child of CLP.

The sample size was determined using the formula, N = z21- α/2 Ϭ2 / E2μ2, and was estimated to be 132. Data collection was scheduled in such a way that one center three days in a week during which three centers were visited each day. Children up to 15 years and those without any systemic disease were included. Parents who were not willing to participate and those who did not provide consent were excluded from the study.

Ethical clearance was obtained from the Institutional Review Board (IRB) of Sri Sai College of Dental Surgery, Vikarabad, with IRB number "609/SSCDS/IRB-E/2017." Prior permission from the medical superintendents of all the concerned hospitals was obtained before the start of the study.

The study period was four months, i.e., from December to March of 2019 and then compiled and checked for completeness. The analysis was done using the Statistical Package for Social Sciences 25.0 (IBM Corp., Armonk, NY). Descriptive statistics and chi-square tests were used.

## Results

A total of 136 subjects participated in the study whose level of social stigma was assessed out of which 37% (50) were males and 65% (86) were females (Tables 1-2).

**Table 1:** Distribution of the parent’s myths and their responses toward the cause of cleft lip and palate

S. No	Questions	Agree	Don’t Know	Disagree
1	Evil spirits	0(0%)	48(35.3%)	88(64.7%)
2	Ancestors’ punishment related to wrongdoing by family	0(0%)	51(37.5%)	85(62.5%)
3	Act of God/fate	49(36%)	34(25%)	53(39%)
4	Pregnant women going out during an eclipse	27(20%)	42(29.7%)	67(49.3%)
5	Pregnant women going out on an auspicious day	5(3.7%)	51(37.5%)	80(58.8%)
6	Holding sharp objects such as knives, scissors, or needles during pregnancy	55(40.4%)	33(24.3%)	48(35.3%)
7	Consanguineous marriage	22(16.2%)	5(3.7%)	109(80.1%)

**Table 2:** Distribution of subjects based on the stigma mean scores

Level of stigma	Number	Percentage
AFFILIATE STIGMA MEAN SCORE	43.25 (1.92)	
LOW	47	34.5%
HIGH	89	65.5%

 A majority of them belonged to the lower-middle class (35.3%) and upper-lower class (45%) (Table 3).

**Table 3:** Distribution of study subjects based on socioeconomic status

Socioeconomic status	Number	Percentage
Upper	5	3.7
Upper Middle	18	13
Lower Middle	48	35.3
Upper Lower	61	45
Lower	4	3
Total	136	100

Of the subjects, 11.8% had positive family history while 88.2% had a negative family history of CLP (Table 4).

**Table 4:** Distribution of study subjects based on family history

Family History	Number	Percentage
Yes	16	11.8
No	120	88.2
Total	136	100

On comparing the order of the child, the majority were first (46%) and second (43%) in the order of their births (Table 5).

**Table 5:** Represents the association of various variables with the Affiliate Stigma score

Factors	Categories	Number/ Percentage	P-Value
Gender of the parent	Male	50(36.8%)	0.30
	Female	86(63.2%)	
Gender of the child	Boys	73(53.7%)	*0.05
	Girls	63(46.3%)	
Socioeconomic status	Upper	5(3.7%)	0.09
	Upper Middle	18(13%)	
	Lower Middle	48(35.3%)	
	Upper Lower	61(45%)	
	Lower	4(3%)	
Order of the child	1	63(46.3%)	0.78
	2	58(42.6%)	
	3	15(11%)	
Family history	Yes	16(11.8%)	0.39
	No	120(88.2%)	
Clinical diagnosis	Cleft Lip	15(11%)	0.87
	Cleft Involving Lip and Alveolus	18(13.2%)	
	Cleft Palate	16(11.8%)	
	Cleft Lip and Palate	87(64%)	

## Discussion

CLP is a relatively common anomaly among Asian populations and is a multi-factorial congenital disorder. Therefore, it requires a multi-disciplinary approach where a dentist plays a key role from the time of birth till adulthood [6-7]. A child is many times, especially in India, considered a gift of God and is even named after a multitude of Gods! Age is an important indicator for the treatment of CLP. We found that 64% of the children were below three years of age, which is similar to a study done by Gopinath VK et al. The Smile Train conducts various campaigns to raise public awareness for CLP extended to remote areas. Hence, young children are brought to these hospitals at the earliest [8].

Forty-six percent (46%) of the children were girls while 54% of them are boys, similar to a study done by De Vries et al. where 61% were females and 39% were males. It suggests that there is a demographic variation of the prevalence of CLP among males and females [9]. Also, India is a country where female feticide is still prevalent. There was a statistically significant association between the Affiliate Stigma scale and gender, which was consistent with the findings of a study done by Patra et al. where higher stigma scores were seen among parents having a girl child with a developmental disability [10]. The reason stated by the author was high face concern, i.e., the value placed over the social position.

The socioeconomic status revealed that 48% belonged to the middle class, which was in accordance with the findings of a study done by Nagappan et al. where 45.5% belonged to the middle class [11-12]. The hospitals included in the present and the above study were non-profit organizations where the majority of those belonging to the disadvantaged classes use these services.

In the current study, 64% of the children were found to have CLP, which was similar to another study done by Fathy et al. where 48% of the children had a cleft involving the palate, 30% had only a cleft lip, and 22% had a cleft involving both cleft lip and palate [13]. This being a hospital-based study and CLP requiring multiple follow-up visits requiring long-term treatments could have resulted in higher recruitment of this type of defect.

Culture-based health beliefs exist in every society concerning the causes of birth defects and genetic disorders. Since culture shapes thinking, one can understand how it can be deeply ingrained into beliefs [14]. Around 36% of the parents have ascribed the cleft to ‘‘God’s will,” which was consistent with the findings of a study done by Oginni et al. in Nigeria [15]. It was attributed in the later study that this belief could be a deterrent for seeking treatment, as it was believed that repair of the cleft would interfere with God’s will. While the relative disagreement in the present study gives a sense of passive acceptance and fortitude and can be broadly considered an act of the Divine with regard to cleft lip and palate.

Family members may experience strong emotions such as guilt, anxiety, and sadness. In the current study, 95% of the parents have disagreed that they felt embarrassed to have a child with the deformity, which was in contrast to the findings of a study done by Bonsu et al. where mothers reported an experience of embarrassment because of the child’s condition [16]. This can be attributed to high awareness regarding CLP in society. Also, the latter study was done in Africa; differences in socio-cultural contexts where the causes of CLP have been associated with the curse of God or bad luck might have resulted in embarrassment and avoidance in their family.

In the present study, 63% of parents have agreed that they felt sad and helpless about having a child with the deformity, which was similar to Nguyen et al. where 61% of the parents felt sad about having the child. It was perhaps because they were upset at the defect and felt helpless to change the abnormal condition of their child [17].

Public gazing or public staring at the child is a usual stigmatizing experience that parents often encounter. It occurs in informal settings such as family gatherings, social functions, religious festivals, and public transport.

On some occasions, parents choose to conceal their child from public appearance in an attempt to avoid public gazing. They may internalize the blame, and they make an attempt to avoid chances of public blame and shame. The attempt of the extended family members to restrict the presence of a child with a disability from public space appears to be a reaction in anticipation to the same.

In the current study, only 10% of the parents agreed that they avoid going out with the child, and around 17% have agreed that they do not dare to tell others that they have a child with a disability, which was in contrast to a study done by Bonsu et al. and Mzezewa et al. where the children were hidden away from the public, and the mothers isolated themselves socially to avoid gossips while some of them relocated to a new community to avoid family and friends’ visits. The acceptance of these children could possibly be due to the cultural disparity [16,18].

In the current study, 65.5% had high stigma scores with a mean score of 1.96, which was in contrast to a study done by Werner et al. where the scores of caregivers’ Affiliate Stigma score was comparatively low (1.72). A straightforward explanation may be that, indeed, these families were not suffering deeply from internalized stigma [19].

Limitations of the study

The first limitation is that the cross-sectional nature of the study could not establish a causal relationship. Second, the results would have been diverse if it was done in a community-based setting using a mixed-method approach, i.e., qualitative and quantitative methods. Therefore, we would also recommend conducting more studies relating to the perceived social stigma by the child, which would, in turn, have an emotional impact on their parents.

## Conclusions

It is a one-of-a-kind study, as there were very limited studies conducted in India measuring the Affiliate Stigma. Cultural factors and myths were also predominant in the study. In addition, the associated social stigma of CLP is a major obstacle for modern treatment. Early understanding among parents and caretakers regarding the need for multiple surgical interventions for their cleft child and constant follow-up and clinic visits is imperative to ensure compliance with treatment.

Hence, we would like to endorse that the government should initiate and supervise public intervention programs in this regard so that parents and children have a healthier and improved quality of life.
